# Triple coding in human SRD5A1 mRNA

**DOI:** 10.21203/rs.3.rs-5390104/v1

**Published:** 2024-12-19

**Authors:** Martina M. Yordanova, Conor Slattery, Mirriam Baranova-Gurvich, Manon Engels, Oscar Ting, Michał Świrski, Jack A. S. Tierney, Håkon Tjeldnes, Jonathan Mudge, Gary Loughran, Dmitry E. Andreev, Pavel V. Baranov

**Affiliations:** University College Cork; University College Cork; University College Cork; University College Cork; University College Cork; University of Warsaw; University College Cork; University College Cork; European Bioinformatics Institute; University College Cork; Shemyakin-Ovchinnikov Institute of Bioorganic Chemistry, RAS; University College Cork

**Keywords:** Overlapping genes, translation initiation, translon, SRD5A1, uORF, ribosome decision graphs, translation control, protein synthesis, gene annotation

## Abstract

**Background:**

Nucleotide sequence can be translated in three reading frames from 5’ to 3’ producing distinct protein products. Many examples of RNA translation in two reading frames (dual coding) have been identified so far.

**Results:**

We report simultaneous translation of mRNA transcripts derived from *SRD5A1* locus in all three reading frames that result in the synthesis of long proteins. This occurs due to initiation at three nearby AUG codons occurring in all three-reading frame. Only one of the three proteoforms contains the conserved catalytical domain of SDRD5A1 produced either from the second or the third AUG codon depending on the transcript. Paradoxically, ribosome profiling data and expression reporters indicate that the most efficient translation produces catalytically inactive proteoforms. While phylogenetic analysis suggests that the long triple decoding region is specific to primates, occurrence of nearby AUGs in all three reading frames is ancestral to placental mammals. This suggests that their evolutionary significance belongs to regulation of translation rather than biological role of their products. By analysing multiple publicly available ribosome profiling data and with gene expression assays carried out in different cellular environments, we show that relative expression of these proteoforms is mutually dependent and vary across environments supporting this conjecture. A remarkable feature of triple decoding is its resistance to indel mutations with apparent implications to clinical interpretation of genomic variants.

**Conclusion:**

We argue for the importance of identification, characterisation and annotation of productive RNA translation irrespective of the presumed biological roles of the products of this translation.

## Background

The redundancy of the genetic code allows for more than one protein sequence to evolve within the same stretch of nucleotides. This property allows to maximize protein coding information content in the confined genomic space of small genomes e.g., those of viruses and bacteria (Johnson and Chisholm 2004; Schlub and Holmes 2020; Normark et al. 1983). Coding in more than one reading frame might appear as an unnecessary complication in larger genomes as its necessity is not imposed by genomic size limitations (Liang 2010). Nevertheless, dual decoding in the human genome is far from an exception (Liang 2010; Liang and Landweber 2006; Michel et al. 2012; Veeramachaneni et al. 2004). Most examples of overlapping coding regions occur in alternatively spliced transcript isoforms where the same exon is decoded in two different reading frames (Liang and Landweber 2006). For example, alternative first exon utilisation in the INK4a/ARF locus results in the translation of two overlapping frames in the second exon and enables the encoding of two distinct proteins, both with tumour suppressor activity (Sharpless 2005).

Alternative translation mechanisms account for a small but growing number of reported dual coding cases. Alternative start site selection in a long transcript isoform from the GNAS locus was shown to result in the synthesis of two functionally related proteins, XLαs and ALEX, from overlapping reading frames in rat (Klemke, Kehlenbach, and Huttner 2001). Initiation at a CUG codon supports translation of an overlapping alternative reading frame to generate POLGARF from *POLG* mRNA (Loughran et al. 2020).

Given that genomic space is not a constraint in eukaryotes with large genomes, what are the benefits of encoding proteins in alternative frames of the same RNA molecule? Encoding functionally related proteins in the same locus can enable coordinated expression and response to certain conditions (Sharpless 2005). Also, it could be easier for a new protein to evolve on an mRNA that is already highly expressed than elsewhere in the genome (Chung et al. 2007).

Overlapping protein coding regions apply specific constraints on sequence evolution and previous studies have assessed certain sequence properties for the identification of dual coding regions (Chung et al. 2007). This phenomenon has been suggested to have an evolutionary advantage under conditions of a high mutation rate through superposition of critical points, reducing their amount in the genome (Peleg et al. 2004). Dual coding regions were also proposed to have a role in the appearance of novel intrinsically disordered regions with new functions (Kovacs et al. 2010).

The past decade has seen advances in technologies such as the ribosome profiling that allowed us to obtain an unprecedented high-resolution map of translated regions in the transcriptome (Ingolia et al. 2009). As a result, we can now observe the expression of overlapping regions that were not predicted by sequence analysis alone such as those evolved most recently e.g., specific to humans.

Using publicly available aggregated ribosome profiling data (Kiniry et al. 2021; Michel et al. 2018, Świrski et al. in preparation) we discovered that the same sequence in *SRD5A1* mRNA is translated into three long protein products from all three reading frames. This occurs due to initiation at three nearby AUG codons in three different reading frames. By placing gene expression reporters under the control of each individual AUG codon we were able to confirm and characterize this, hitherto unique, example of triple decoding in humans. Strikingly, it appears that the most efficient translation initiation site is an AUG codon not annotated as start. Translation initiated at this start codon produces a protein with yet unknown function. A remarkable feature of triplet decoding is its resistance to frameshifting mutations in the presence of which the catalytic proteoform remains to be produced from one of alternative start codons.

## Results

### SRD5A1 locus and its annotation

**Figure 1:**
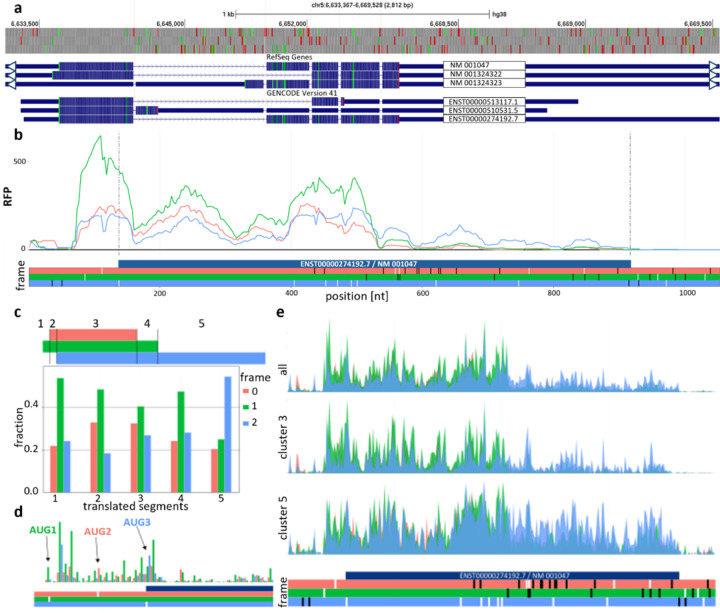
Aggregated Ribo-seq data track provides evidence for translation in three frames. **a** Top: open reading frame (ORF) plot with AUG codons in green and stop codons in red. RefSeq and GENCODE *SRD5A1* transcript annotations. Coding and non-coding exon regions are indicated with blue panels of different thickness, introns with thin lines. **b** Ribosome footprint density from aggregated data tracks in RiboCrypt for Ensembl transcript ENST00000274192.7 corresponding to Refseq transcript NM_001047. The line colouring matches the best supported reading frame in the ORF plot below; start codons shown as white bars, stop codons are black. **c** Frame distribution of Ribo-seq reads mapping to regions of relative overlap between the 3 translon’s ORFs. **d** Sub-codon footprint density where in-frame peaks for all 3 AUG codons indicate active translation initiation. Density peaks are coloured to match the best supported reading frame below. **e** Sub-codon coverage plots for two distinct clusters and all samples combined showing differential translation of translons between clusters.

Steroid 5-alpha-reductase 1 encoded by *SRD5A1* is involved in the regulation of androgen metabolism and is of great interest in the fields of endocrinology and oncology. SRD5A1 converts testosterone into the more potent androgen dihydrotestosterone (DHT). Increased levels and activity of SRD5A1 in prostate cancer result in high DHT levels and castration-resistant prostate cancer (Chang et al. 2011). SRD5A1 activity is also elevated in other sex hormone related cancers (Lewis, Wiebe, and Heathcote 2004; Sinreih et al. 2015; Tanaka et al. 2015). The *SRD5A1* locus has 6 exons whose alternative splicing gives rise to several transcript isoforms annotated by RefSeq (O’Leary et al. 2016) and GENCODE (Frankish et al. 2023) annotations (Fig. 1a). Exon 1 is shared by all annotated transcript isoforms and in it there are 4 AUGs (here and further for simplicity and uniformity we use AUG irrespective whether we refer to sequences of RNA or DNA due to their significance at RNA level). Synthesis of the major annotated SRD5A1 proteoform of 259 AA residues derives from translation initiation at AUG3 in transcript NM_001047/ENST00000274192.7. An alternative proteoform of 212 AA residues with the same C-terminus, is predicted to originate from translation initiation at AUG2 in transcript NM_001324322 lacking exon 3. In transcript NM_001324323, the annotated initiation codon is in exon 2 where it is found downstream to 20 other AUGs raising the question how the scanning ribosomal complexes could reach this AUG. In the absence of experimental evidence and plausible mechanisms explaining translation initiation at this AUG we did not consider it in our study. In all GENCODE transcripts AUG3 is annotated as the sole initiation site (Fig. 1a).

### Aggregated ribosome profiling data suggest predominant translation at AUG1

Ribosome profiling data aggregated in GWIPS-viz (Michel et al. 2018), Trips-Viz (Kiniry et al. 2021, Świrski et al. in preparation) confirmed expression of the AUG3 initiated translon (translated region) encoding the major SRD5A1 proteoform as well as that of the AUG2 initiated translon. Fig. 1b shows a screenshot of aggregated data from 2783 datasets available in RiboCrypt. The data indicate that the translation initiated at AUG1 is predominant as the majority of footprints support its reading frame (green) from its start to its stop (segments 1–4 in Fig. 1c). Translation initiation at AUG3 is also evident since footprints downstream of AUG1 initiated translon (non-overlapping segment 5 in Fig. 1c) predominantly support its frame (blue). Detection of AUG2 initiated translon is the hardest because it overlaps with the other two near-entirely. However, the data do support its translation because footprints in the area where all three translons overlap (segment 3 in Fig. 1c) have higher support for its frame than for that of AUG3 (red over blue), while downstream of the AUG2 translon (segment 4 in Fig. 1c) footprints supporting AUG3 frame dominate over AUG2 frame (blue over red). This switch likely occurs because of termination of ribosomes translating AUG2 translon (red). Fig. 1d provides a nucleotide resolution of footprint density for the area of three AUG codons which is also consistent with simultaneous usage of all three AUGs as start codons. It can be seen that the highest peaks of inferred P-site codons at these AUG codons match their respective frames.

Translation machinery varies across cell types (Anisimova et al. 2023) and conditions, and translation is particularly sensitive to stress (Andreev et al. 2015; Costa-Mattioli and Walter 2020). To examine whether relative synthesis of the three different proteoforms changes across cells and conditions we analysed of 2783 Ribo-seq libraries (Additional file 1) and clustered them based on relative translation of 5 segments depicted in Fig. 1c (Methods). This led to the identification of five distinct data clusters (Fig. 1e and Additional file 2: Fig. S1). Cluster 5 is characterised by elevated relative translation of the AUG3 initiated translon. This cluster comprises of 29 Ribo-seq libraries derived from 8 studies representing three cell lines: HeLa (12 samples), PC3 (7 samples), MCF (4 samples) (Additional file 1). The remaining two samples are from human liver tissue (Additional file 1). Of note, all 7 PC3 libraries in cluster 5 originated from the same study (Loayza-Puch et al. 2016). In our dataset there is only one other study featuring PC3 cells (Hsieh et al. 2012) however the coverage was insufficient and did not pass our threshold for expression levels. The enhanced AUG3 translon expression observed in cluster 5 (samples from breast, ovary and prostate cancer cell lines) is consistent with previously observed elevated SRD5A1 activity in sex hormone related cancers (Lewis, Wiebe, and Heathcote 2004; Sinreih et al. 2015; Tanaka et al. 2015).

In addition to analysing distribution of footprints of elongating ribosomes we took advantage of the data obtained with translation inhibitors that selectively arrest initiating ribosomes (Ingolia, Lareau, and Weissman 2011; Ingolia et al. 2012; Lee et al. 2012). This type of data is less quantitatively reliable due to reliance on discrete locations of potential start codons making them prone to sequence biases. Yet, it may be used for qualitative assessments of translation initiation. Out of the 8 initiation studies available in the GWIPS-viz browser, 4 had footprint signals above background levels in the *SRD5A1* 5’ leader, and from these, preferential initiation at AUG1 was revealed for MCF, HCT116 and iPSC cells (Additional file 2: Fig. S2). Only one study, from Jurkat cells, had the largest initiation peak corresponding to initiation at AUG3, providing additional evidence for cell type specific effects on start site selection. Interestingly, translational effects specific to Jurkat cells have been noted previously (Plank, Whitehurst, and Kieft 2013).

### Reporter evidence for triple decoding in the SRD5A1 5’ leader

**Figure 2:**
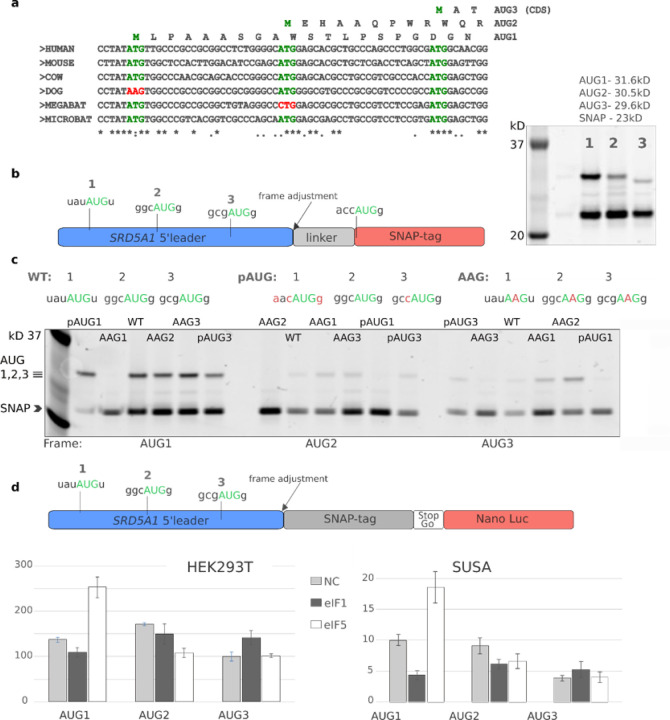
Reporter assay validation of translation initiation at three AUG codons in the *SRD5A1* 5’ leader. **a** An alignment of representative mammalian genomic sequences in the vicinity of the 3 AUGs in *SRD5A1* 5’ leader showing examples of AUG losses. **b** Schematic of the reporter where SNAP-tag encoding sequence was fused downstream to the *SRD5A1* 5’ leader sequence. Binding of SNAP-tag substrate allows visualisation directly in the protein gel. Gel on the right shows all three frames are translated. **c** Initiation at each AUG codon was monitored in the presence of selected nucleotide mutations, pAUG denotes perfect Kozak context. **d** eIF1/eIF5 overexpression to assess the stringency effect on start codon selection in *SRD5A1* 5’ leader in HEK293A and SUSA cell lines; plotted are normalised NanoLuc activities. **Top**: schematic of the construct where NanoLuc activity reports on each of the frames expression.

The nucleotide sequence in the vicinity of three AUG codons is shown in Fig. 2a along with sequences of other mammals. To examine AUG utilisation by an orthogonal approach, we generated reporter constructs where the full-length 5’ leader sequence of the canonical *SRD5A1* transcript (ENST00000274192.7/NM_001047), followed by the first ten codons downstream of AUG3, were fused upstream of the SNAP-tag encoding sequence (Fig. 2b). Initially, three *SRD5A1*-SNAP constructs were generated, with the SNAP-tag encoded in each of the three reading frames. Detection of SNAP-tag-containing products following transient transfection of HEK293A cells confirmed the evidence from ribosome profiling data, namely that ribosomes initiate translation at all three AUG codons (Fig. 2b, right). Moreover, the intensity of the bands suggested that AUG1 was the most efficiently utilized, followed by AUG2 and AUG3.

The occurrence of a SNAP-only band of approximately 23 kD (Fig. 2b) most likely resulting from initiation at the SNAP-tag AUG in a perfect Kozak context (Kozak 1986; Noderer et al. 2014), suggested a highly efficient leaky scanning of the *SRD5A1* 5’ leader. However, it would be misleading to assume that the high intensity of this SNAP-only band solely reflects the number of ribosomes reaching the SNAP AUG codon. It is likely that the SNAP-only protein product is more stable or exhibits a greater affinity for the SNAP-tag substrate compared to the N-terminally extended proteoforms, (Bachmair, Finley, and Varshavsky 1986). Indeed, the difference in band density between SNAP-only and the 5’ extended SNAP proteoforms was less pronounced when proteasome activity was inhibited by MG 132 (Additional file 2: Fig. S3).

To validate that the observed bands originated from initiation at each of the three AUG codons, specific nucleotide substitutions were introduced. Changing each AUG codon to an AAG resulted in an anticipated loss of the corresponding band on the gel. Mutating AUG1 to AAG led to an expected increase in initiation at the two downstream AUG codons, while initiation at AUG3 was also enhanced by AUG2 to AAG substitution. These findings imply that, as predicted by the leaky scanning model of translation, the expression of AUG3 could be regulated by modifying the efficiency of initiation at the upstream AUGs (Fig. 2c). We also improved the Kozak context of AUG1 and assessed the expression of each AUG in the presence of alterations to the other two AUG codons. The perfect context of AUG1 eliminated the band corresponding to initiation at AUG2, while initiation at AUG3 was still detectable arguing that some scanning ribosomes continue through the first two AUGs even when placed in the perfect context (Fig. 2c).

To enable more precise quantification of expression in the three reading frames, we designed constructs where NanoLuc activity reports separately on the translation of each frame. In these constructs, NanoLuc was fused to SNAP-tag (Fig. 2d). Placing the StopGo (also known as 2A) (Atkins et al. 2007; Donnelly et al. 2001) sequence between that of SNAP-tag and NanoLuc aimed to neutralize the effects of different N-termini on reporter activity. The luciferase assay revealed similar levels of expression for the AUG1 and AUG2 initiated frames, followed by AUG3 (Fig. 2d, **light grey bars**).

The recognition efficiency of start sites, particularly those with weak Kozak contexts like AUG1, is influenced by the levels of specific translation factors, such as the stringency factors eIF1 and eIF5 (Ivanov et al. 2010; Loughran et al. 2012). It has been shown that the levels of these and other translation factors varies between different cell types, thereby creating distinct cellular environments affecting translation (Anisimova et al. 2023).

To investigate the impact of eIF1 and eIF5 levels on the stringency of start site selection in the *SRD5A1* 5’ leader, we examined the NanoLuc reporter constructs in cells co-transfected with eIF1 or eIF5 expressing vectors (Fig. 2d and Additional file 2: Fig. S4, **dark grey bars**). Consistent with its role in enhancing the stringency of start site selection (Loughran et al. 2012), elevated levels of eIF1 led to a reduced initiation at AUG1 in a poor Kozak context. The effect was much more pronounced in the testis germ cell line SUSA possibly reflecting differences in endogenous eIF1/eIF5 levels. A concomitant increase in AUG2 and AUG3 expression was more evident in HEK293T cells (Fig. 2d and Additional file 2: Fig. S4). Upon overexpression of eIF5 which reduces the stringency of start site selection, a nearly twofold increase in AUG1 expression was observed, while expression of AUG2 and AUG3 remained largely unchanged.

### Ribosome decision graphs of SRD5A1 transcripts

Our experimental data in combination with publicly available ribosome profiling data demonstrated that all three AUG codons serve to initiate translation in three overlapping frames in the *SRD5A1* 5’ leader. AUG1 is in a poor Kozak context (uauAUGu) which supports efficient leaky scanning allowing for the downstream AUGs to be initiated. Most current transcript annotations indicate AUG3 as the sole translation initiation site. In transcripts NM_001324322 and NM_001324323, AUG2 and an AUG from the second exon are annotated as initiation sites, respectively, the rational most likely being that initiation at these codons would support the synthesis of the longest and catalytically active proteoforms from each transcript.

**Figure 3:**
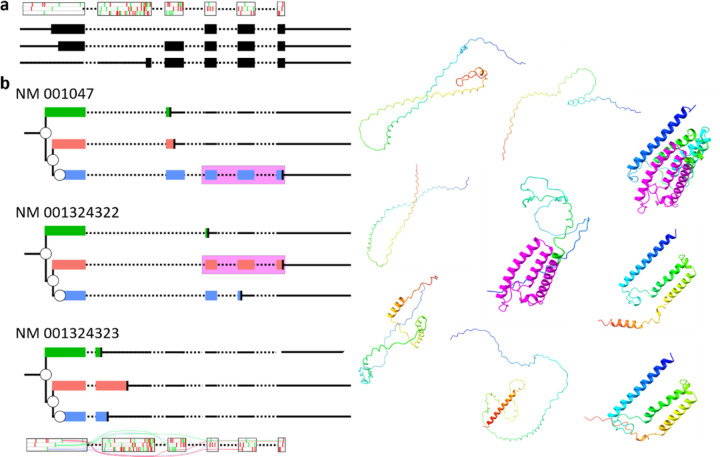
Representation of translation at three *SRD5A1* RNA transcripts. **a**Three RefSeq *SRD5A1* transcript isoforms all sharing the first exon but with different AUG codon annotated as a start site. Above is a schematic of the ORF organisation for each coding exon with AUG codons in green and stop codons in red. **b** RDG representations for each of the three transcripts with putative translons indicated; right: AlphaFold2 structures for each of the predicted proteoforms. Regions of the translons highlighted in purple indicate encoded SRD5A1 enzymatic activity. Rainbow colouring shows N-terminus (blue) to C-terminus (red) orientation.

However, since all transcript isoforms share exon 1, and there is no evidence that the selection of start codons by the ribosomes depend on the exon organization downstream, the scanning ribosome will be similarly likely to initiate at an AUG codon in each of the transcript isoforms. The resulting products of translation will depend on the downstream exon organisation. To illustrate the transcript specific translation paths, we took advantage of Ribosome Decision Graphs (RDGs) (Tierney et al. 2023) (Fig. 3b). Informed by our experimental data, RDG representations predict the synthesis of at least three protein products from each transcript isoform.

### Phylogenetic analysis

Comparative sequence analysis provided support for the ENST00000274192.7/NM_001047 transcript being the ancestral form, at least with respect to the mammalian common ancestor. All three AUGs are likely ancestral to placental mammals, see genomic sequence alignments in Fig. 2a and Additional file 2: Fig. S5. From an evolutionary perspective, it is striking that the CDS in the first exon of this transcript is highly divergent within mammals, especially over the first ~50 aa. Even just between human and mouse this region is hard to align suggesting that the protein is highly dynamic in evolutionary terms. This would seem to fit with the overall narrative about the function of the gene (*SRD5A1* being involved in testosterone processes, with the role of testosterone in mammals itself apparently being subject to rapid changes (Husak et al. 2021).

Although all three AUG codons seem ancestral, independent losses of AUG1 and AUG2 are observed in multiple lineages in placental mammals (Fig. 2 and Additional file 2: Fig. S5). Furthermore, stop codons interrupting their ORFs (relative to human) are even more frequent across different species, suggesting weak selection (if any) on the products of their translation initiation. Given the comparatively high conservation of these AUGs and the knowledge that AUGs in 5’ leaders are generally under negative selection (Churbanov et al. 2005; Whiffin et al. 2020), it is reasonable to infer their functionality. It is likely that their evolutionary significance lies in the regulation of *SRD5A1* catalytic proteoform expression rather than in the products of their own expression. Nonetheless we could not exclude the possibility of a functional significance of non-catalytic proteoforms in humans and closely related primates.

## Discussion

We present the first documented case of triple decoding in a human gene, where three long overlapping open reading frames are translated simultaneously from the same mRNA—a phenomenon previously considered only a theoretical possibility. These translons result in the synthesis of at least three different protein products from each *SRD5A1* mRNA, as illustrated with ribosome decision graphs (Fig. 3b). Only one of the three proteoforms translated from one of the two transcripts (AUG2 or AUG3) contains a catalytical domain of steroid 5-alpha-reductase, while the biological significance of the other seven proteoforms remains unclear.

Most surprisingly, both ribosome profiling and expression reporter evidence indicate that AUG1 is by far the most efficient initiation codon, despite not producing proteoforms with a catalytic domain from any of the annotated transcripts. We have not yet explored the functional significance of alternative proteoforms lacking the catalytic domain. Given the absence of evidence for evolutionary purifying selection, the AUG1-initiated proteoforms may not have specific biological roles beyond potentially contributing to the pool of immunopeptides.

Nonetheless, our data suggest that the translation of these proteoforms modulates the synthesis of catalytic proteoforms, as we observed differential translation of the same RNA reporters in different cell lines (HEK293T and SUSA). Additionally, we demonstrated that altering the stringency of start codon selection can influence the relative synthesis of these proteoforms. Since AUG1 is in a poor Kozak context, reduced stringency increases its efficiency, thereby repressing the synthesis of catalytic proteoforms initiated from downstream AUGs. By analysing thousands of available ribosome profiling datasets, we have also identified differential translation of these translons across various human samples. This variation may be due to differences in the abundance of translation factors or the composition of RNA-binding proteins interacting with *SRD5A1* mRNA. Our study highlights the utility of large-scale Ribo-seq data reanalysis in characterizing differential translation in varying cellular environments.

The surprising finding that the unannotated proteoform is synthesized at the highest rate raises questions for future genome annotation efforts. To date, annotation has focused on identifying protein-encoding sequences under purifying selection using codon substitution models (Lin, Jungreis, and Kellis 2011) as the gold standard. Purifying selection acts on specific genotypes due to their positive effects on fitness, unambiguously indicating the functional significance of the encoded feature. However, the advent of ribosome profiling, which provides an unbiased assessment of protein synthesis at the whole-cell level, has revealed widespread translation beyond the regions annotated as protein-coding (Chew et al. 2013; Ingolia et al. 2014). The significance of these enigmatic translons, also known as RiboSeq ORFs, is currently under investigation (Mudge et al. 2022). It is likely that a substantial fraction of these translons arises due to mechanistic requirements of translation initiation process rather than adaptive evolution, explaining the lack of evolutionary selection acting on their product sequences. Nonetheless, they result in the production of a significant number of protein molecules that may potentially affect biochemical processes in our cells, with either positive or negative consequences. The example of *SRD5A1* illustrates that, in certain loci, the translation of such enigmatic translons could exceed the translation of those encoding proteins under selection.

Furthermore, our finding emphasises the need to annotate productive translation irrespective of the functional role of the protein products for appropriate interpretation of genomic variants. Currently it is assumed that an indel mutation occurring in a protein coding region would be deleterious for its expression and function because of premature termination in the shifted reading frame. However, any indel mutation within the overlapping region of three translons would not be deleterious for the expression of the catalytic *SRD5A1* proteoform, since it will always be produced from one of the three AUG codons in any of the frames. Moreover, a single nucleotide deletion would place the catalytic domain in-frame with AUG1, thus likely increasing the levels of *SRD5A1* expression. The resistance of gene expression to indels in the coding regions is not limited to triple decoding, either a deletion or an insertion of a nucleotide is expected to retain expression of a gene in case of dual decoding which is frequent in humans.

## Conclusions

*SRD5A1* locus contains a long segment translated in all three reading frames into three long protein products. This organisation of protein coding information allows for differential regulation of translation in different cellular environments and also provides resistance of SRD5A1 catalytical domain expression to indel mutations in the region where three translons overlap.

## Methods

### Data analysis

Per-sample (2783 libraries) RiboCrypt P-site coverage tracks for SRD5A1 locus were used for the clustering and translation detection evidence (Fig. 1b–e & Additional file 2: Fig. S1.) (Świrski et al. in preparation). Clustering of entire transcript-level coverages was performed with the unsupervised k-means algorithm into 5 clusters, a functionality available under RiboCrypt metabrowser utility (https://ribocrypt.org/#MetaBrowser). Relative translation analysis of the three overlapping translons (Fig. 3 c–d) was done by summing P-site coverage across all libraries for 5 unique overlaps between the 3 translons: Just translon 1, translon 1 + 2, translon 1 + 2 + 3, translon 2 + 3, translon 3.

Genomic sequence alignments were visualised and explored with CodAlignView (Jungreis et al. 2021) using 100-way vertebrate genomic alignment (Rosenbloom et al. 2015) with human genome hg38 assembly as a reference.

### Cloning and sequences

Primers were synthesized by IDT, and their sequences are listed in (Additional file 3). The primers were designed to amplify the sequence of the entire *SRD5A1* 5’ leader (as in MANE transcript) until and including 10 codons downstream of AUG3. This sequence was generated by a polymerase chain reaction (PCR) using as a template genomic DNA extracted from HEK293A cells. The test sequence was cloned (SacI + XbaI) (New England Biolabs (NEB)) directly following that of the CMV promoter in pcDNA3.4 vector expressing SNAP-tag. The last 40 codons from hemoglobin beta (*HBB*) coding region were used as a linker between the *SRD5A1* sequence and that encoding SNAP-tag. Expression of the three reading frames was monitored in three separate constructs, where SNAP-tag was placed in alternative reading frames by inserting one or two nucleotides following the 5’ leader sequence. Mutations were generated using in vivo assembly (IVA) cloning as described in (García-Nafría, Watson, and Greger 2016). In the constructs used for the luciferase assay, StopGo-NanoLuc encoding sequence, obtained as gene block from IDT, (Additional file 3) was introduced in place of the SNAP-tag stop codon.

### Tissue culture and cell transfection

Human Embryonic Kidney 293A (HEK293A) cells (ATCC), Human Bone Osteosarcoma Epithelial Cells (U2OS) (ATCC), and Human Prostate Adenocarcinoma Epithelial Cells (PC-3)(ATCC), were maintained as monolayer cultures, grown in Dulbecco’s modified Eagle’s medium (DMEM), (Sigma-Aldrich) supplemented with 10% foetal bovine serum (FBS), 1% penicillin–streptomycin and 1% L-glutamine (Thermo Fisher). Human Testicular Epitheloid Cells (SuSa) (DMSZ) were grown as adherent monolayer cultures in 80% RPMI (Thermo Fisher) media supplemented with 20% FBS, and antibiotics at 37°C in an atmosphere of 5% CO2.

### Transfection with SRD5A1-SNAP-tag expressing plasmids.

4 × 10^6^ HEK293A, U2OS, SUSA and 2 × 10^6^ PC-3 cells were plated on 10-cm tissue culture dishes. After 24 h, the cells were detached with trypsin, suspended in fresh media, and transfected with Lipofectamine 2000 reagent (Invitrogen), using the one-day protocol in which suspended cells are added directly to the DNA complexes in 24-well plates. For each transfection, the following was added to each well: 200 ng plasmid DNA, 1.3 μL Lipofectamine 2000, and in 200 μL Opti-MEM (Thermo Fisher). Next, 2 × 10^5^ for HEK293A, U2OS, SUSA and 1 × 10^5^ for PC3 in 800 μL DMEM were added to the transfecting DNA complexes in each well. Transfected cells were incubated for 24 h at 37°C in 5% CO2.

### Transfection with SRD5A1-NanoLuc expressing plasmids

Cells were detached with trypsin, suspended in fresh medium, and transfected in four replicates with Lipofectamine 2000 reagent (Invitrogen), using the 1-day protocol in which suspended cells are added directly to the DNA complexes in half-area 96-well plates. For each transfection the following was added to each well: 25 ng of plasmid DNA, 0.2 μl of Lipofectamine 2000 in 25 μl of OptiMem (Gibco). 4 × 10^4^ cells in 50 μl of DMEM were added to the transfecting DNA complexes in each well. Transfected cells were incubated at 37 C in 5% CO_2_ for 20 h and assayed using the Dual-Luciferase assay.

### MG132 treatment

Media was removed from the cells, and media in each well was replaced with 800 μL fresh DMEM supplemented with 10μM MG132 (Sigma) for 5-hour incubations, or 5μM MG132 for a 20 hour incubation. Incubation was at 37°C in 5% CO2.

### Protein isolation and electrophoresis

24 hours after transfection, cells were washed with 1× PBS, and whole-cell lysates were prepared in PLB (Promega) buffer supplemented with SNAP-Cell 647-SiR fluorescent substrate (final concentration 0,01 μM; NEB) and DTT (final concentration 1 mM; Biosearch Technologies). Cell lysates were incubated for 30 min at room temperature with shaking. Lysates were then clarified by centrifugation for 10 minutes at 16,000 x g at 4°C and protein concentrations were measured using the Nanodrop Spectrophotometer (Thermo Fisher). Sample buffer (Bio-Rad Laboratories) supplemented with 5% β-mercaptoethanol was added to all protein lysates and samples were loaded onto a 4–12% Bolt^™^ Bis-Tris Plus Mini Protein Gel (Thermo Fisher). Protein gels were scanned with Typhoon Trio+ instrument (Amersham) using the 670 BP 30 emission filter.

### Dual-Luciferase assay

The luciferase assay buffers were prepared in-house following the protocol described in (Dyer et al. 2000). Relative light units were measured on a Veritas microplate luminometer fitted with two injectors (Turner Biosystems, Sunnyvale, CA). Transfected cells were then lysed in 18 μl of 1× passive lysis buffer (PLB; Promega), and light emission was measured following injection of 50 μl of each luciferase substrate buffer. A firefly-expressing plasmid was co-transfected alongside each *SRD5A1*-NanoLuc plasmid and was used as a transfection control. For each data point NanoLuc activity was normalized relative to the firefly activity. For each cell line, three independent transfection experiments were performed each with 4 technical replicates. The four data points for each construct were averaged, and standard deviations calculated. Although we have not used reporters encoded on the same RNA we followed recently developed MINDR guidelines (Loughran et al 2024) and report absolute readout values (Additional file 4) and list the construct sequences (Additional file 5).

## Supplementary Material

Supplement 1

## Data Availability

All data generated in this study are included in this published article [and its supplementary information files].
