# Supplementary material for: Triple coding in human SRD5A1 mRNA
Source: Res Sq. 2024 Dec 19:rs.3.rs-5390104. Preprint. [Version 1] doi: 10.21203/rs.3.rs-5390104/v1 (PMC11702784; doi:10.21203/rs.3.rs-5390104/v1)
Supplement: Supplement 1 [file NIHPPRS5390104v1-supplement-1.pdf]

Supplementary Files

This is a list of supplementary files associated with this preprint. Click to download.

- Additionalfile1.xlsx
- AdditionalFile2.docx

- Additionalfile3.xlsx
- Additionalfile4.xlsx
- Additionalfile5.docx
